# Immuno-cardio-oncology: Killing two birds with one stone?

**DOI:** 10.3389/fimmu.2022.1018772

**Published:** 2022-11-17

**Authors:** Sophie Van Linthout, Hans-Dieter Volk

**Affiliations:** ^1^ Berlin Institute of Health (BIH) at Charité-University Medicine Berlin, BIH Center for Regenerative Therapies (BCRT), Berlin, Germany; ^2^ German Center for Cardiovascular Research (DZHK), Partner Site Berlin, Berlin, Germany; ^3^ Institute of Medical Immunology, Charité-University Medicine Berlin, Berlin, Germany

**Keywords:** immuno-cardio-oncology, inflammation, immune cell dysbalance, repurposed therapies, patient stratification, interdisciplinarity

## Abstract

Inflammation and a dysregulated immune system are common denominators of cancer and cardiovascular disease (CVD). Immuno-cardio-oncology addresses the interconnected immunological aspect in both cancer and CVD and the integration of immunotherapies and anti-inflammatory therapies in both distinct disease entities. Building on prominent examples of convergent inflammation (IL-1ß biology) and immune disbalance (CD20 cells) in cancer and CVD/heart failure, the review tackles both the roadblocks and opportunities of repurposed use of IL-1ß drugs and anti-CD20 antibodies in both fields, and discusses the use of advanced therapies e.g. chimeric antigen receptor (CAR) T cells, that can address the raising burden of both cancer and CVD. Finally, it is discussed how inspired by precision medicine in oncology, the use of biomarker-driven patient stratification is needed to better guide anti-inflammatory/immunomodulatory therapeutic interventions in cardiology.

## Introduction

Initially focused on the detrimental effects of cancer therapies on the cardiovascular system, the field of cardio-oncology has expanded, and further investigates the commonalities between cancer and CVD. In this regard, there is accumulating evidence that inflammation and a dysbalanced immune system are common triggers in the pathogenesis of cancer and CVD. This concept is covered in immuno-cardio-oncology, which beyond the use of immunotherapies or anti-inflammatory therapies to counteract cancer or cancer therapy-related side effects also addresses the interconnected immunological aspect in both cancer and CVD, and the hereto-related potential of integrating immunomodulatory strategies in both disease entities. Important in this context is the raising appreciation that cancer and CVD/heart failure interact in a bidirectional manner with low-grade inflammation as common trigger. Improved cancer prognosis and survival rate due to the success of recently implemented onco-therapies, allowed the awareness that among survivors, CVD is the leading cause of noncancer-related mortality (1). For lung cancer, it has been reported that 89% of the patients have an increased risk of developing atherosclerotic heart disease compared to those not afflicted with cancer (2, 3). In general, a low-grade chronic inflammation provoked by the release of pro-inflammatory cytokines (tumor necrosis factor-α, interleukin (IL)-1β, IL-6, and interferon-γ), chemokines, and soluble factors by the primary tumor cells and cells of the microenvironment into the bloodstream is suggested to stimulate CVD and heart failure (4). On the other hand, there is epidemiological evidence that CVD patients are more prone to develop cancer (5, 6), stating CVD as an oncogenic risk factor (7, 8). This is further supported by experimental findings illustrating that failing hearts stimulate tumor growth (9–12) (reverse cardio-oncology) independent of hemodynamic impairment (10), *via* epigenetically driving myeloid cells in hematopoietic reservoirs toward an immunosuppressive state and inducing monocytosis (9) and *via* the release of inflammation markers like the matricellular protein periostin (11). The relevance of secreted tumor-promoting factors is further corroborated by the observation that heart failure and inflammation markers are associated with new onset cancer incidence among participants with heart failure (10). This bidirectional interaction of cancer and CVD with inflammation and a dysregulated immune system as common denominators offer the opportunity to transfer knowledge, technologies and concepts from the haema-/oncology field to the cardiovascular field and *vice versa*. It further opens avenues to accelerate the repurposed use of approved anti-inflammatory and immunomodulatory therapies, including advanced therapies e.g. chimeric antigen receptor (CAR) T cells, that can treat both disease entities (13).

With the activation of IL-1ß signaling and dysregulation of CD20 B immune cells being prominent examples illustrating the common involvement of inflammation and a dysregulated immune balance in cancer and CVD, this minireview addresses how further inspired by the CANTOS trial, anti-IL-1ß drugs are beyond CVD and heart failure, evaluated in cancer, and how *vice versa* the anti-CD20 monoclonal antibody (rituximab), the first therapeutic antibody approved for oncology patients, is evaluated in the context of CVD and heart failure. Next, the repurposed use of advanced therapies is discussed, to finally comment the roadblocks and challenges for anti-IL-1ß and anti-CD20 repurposed strategies in cancer and CVD, and the lessons learned for improved therapies, including the relevance of biomarker-driven stratification and treatment of patients.

## Convergence of inflammation and immune cell disbalance in CVD, heart failure and cancer and repurposed therapies

### IL-1ß biology and anti-Il-1ß therapies

The landmark CANTOS (Canakinumab Anti-Inflammatory Thrombosis Outcome Study) trial (14), the largest cytokine inhibition trial ever completed, providing compelling proof for the inflammatory hypothesis in atherothrombosis, further evidenced that inflammation is an important trigger and valuable target in both CVD and cancer. Along with the primary observation that rates of cardiovascular events were lower in canakinumab-treated patients compared to the placebo group, further investigations revealed that IL-1ß antagonism reduced the incidence of lung cancer and cancer-related mortality (15), accentuating convergence in IL-1ß biology in CVD and cancer. Canakinumab treatment did not alter all-cause mortality, which was due to offsetting effects of reduced cancer mortality but increased fatal infections. Finally, the outcome of CANTOS was FDA rejection and European license withdrawal. The full reasons for the rejection have not been disclosed by Novartis, which do not further pursue canakinumab for cardiovascular indications, but test now its anti-cancer potential in patients with no-small-cell lung cancer (16). The high pricing of canakinumab (ca. 200,000 €/year in the United States), further favors its use for (no-small-cell lung) cancer rather than for a common indication as a secondary prevention following myocardial infarction (MI) (17).

IL-1ß is generated by the Nod-like receptor protein 3 (NLRP3) inflammasome, a multiprotein complex, part of the innate immunity, which gets activated following dangerous associated molecular patterns, like the alarmins S100A8 and S100A9 (18, 19), tobacco (20) and cholesterol crystals (21), as well as by pathogen associated molecular patterns (PAMPs), like coxsackievirus B3 (22, 23) and human papillomavirus (24), covering a broad spectrum of triggers present in or provoking both CVD/heart failure and cancer (25). Oxidized LDL and cholesterol crystals are DAMPs which activate the NLRP3 inflammasome and lead to IL-1ß secretion. IL-1ß subsequently drives atherogenesis at different stages. It increases the adhesion and homing of pro-inflammatory monocytes as well as the expression of matrix metalloproteinases, the latter boosting plaque rupture (21). IL-1ß release following MI leads to myelopoiesis and splenic monocytosis (26). Hereby, it triggers the homing and infiltration of monocytes to the atherosclerotic plaque, supporting the so called “cardiovascular continuum” and begetting subsequent MI (27). The CANTOS study was built on this concept and the hypothesis that anti-IL-1ß antagonism could blunt the recurrence of cardiovascular events. Beyond atherosclerosis and ischemic heart disease, the NLRP3 inflammasome and downstream cytokines IL-1ß, IL-18, to IL-6 play a key pathogenic role in non-ischemic, inflammatory heart disease [myocarditis (22, 28–30), pericarditis (31)] and the progression to heart failure (32, 33).

Evidence states that polymorphisms in the *NLRP3* inflammasome gene are linked with CVD and cancer development (25). Clonal hematopoiesis, the occurrence of recurrent somatic mutations in leukemia-associated genes, most commonly in *DNMT3A, TET2, and ASXL1*, promoting expansion of clonal populations of hematopoietic stem or progenitor cells, is associated with hematologic malignancies like acute leukemia and can also occur in the absence of overt hematologic transformation. In fact, the latter, so called clonal hematopoiesis with intermediate potential (CHIP), contributes causally to the development of CVD and doubles the risk for CVD, whereas clonal hematopoiesis only accounts for 0.5% of hematologic cancers (34, 35). Intriguingly, loss of the *TET2* gene in hematopoietic cells, encoding an epigenetic regulatory protein involved in DNA methylation, accelerates atherogenesis involving increased NLRP3-mediated IL-1ß signaling (36). In addition, an explorative study demonstrated that presence of CHIP variant *TET2* clones may predispose patients to improved outcomes with targeted anti-IL-1ß therapy (37). Clonal hematopoiesis is an age-dependent risk factor for leukemia and CVD and can occur without candidate driver mutations (38). It is further common in patients with non-hematologic cancers following radiation or chemotherapy where it is associated with an increased risk of hematologic cancers and adverse clinical outcome (39).

In cancer, NLRP3 and IL-1ß drive cancer progression by different means, involving promotion of tumorigenesis, angiogenesis, immunosuppression, and metastasis (40). NLRP3, IL-1ß and downstream IL-6 are further activated following cancer therapies including doxorubicin (41–44), tumor cell-targeting CAR T cells (45, 46) and immune checkpoint inhibitors (47, 48), contributing to the cytokine release syndrome and cardiac toxicity. The relevance of NLRP3 and IL-1ß in cancer progression and cancer therapy-related (cardiac) detrimental effects form the rationale of several clinical trials currently investigating the efficacy of anti-IL-1ß drugs, mainly the IL-1ß antagonist canakinumab and the natural anti-ILR antagonist, anakinra, as anti-cancer therapy alone, or in combination with CAR T cells or the checkpoint inhibitors anti-PD1 or anti-PDL1 (40). Clinical trials directed to investigate the anti-cancer effect of specific NLRP3 inflammasome inhibitors which either target components of its canonical signaling pathway or are specific to the NLRP3 protein, have not been performed so far (49). This might be explained by the complexicity of NLRP3 in cancer and stresses the need for further preclinical studies. Indeed, beyond tumorigenic effects, also anti-tumorigenic effects of the NLRP3 inflammasome have been reported depending on the type of cancer (49). This involves among others the NLRP3-mediated release of IL-18 and subsequent promotion of natural killer cell tumoricidal activity (50, 51). The tumor-suppressive function of the NLRP3 inflammasome has mostly been demonstrated for colon cancer where its preventive role is achieved by tumor immunosurveillance, maintaining epithelial integrity, producing mucus and suppressing the proliferation of intestinal epithelial cells (52). It is the result of cell type-specific responses, which altogether determine the propensity for tumorigenesis in colon cancer (53). Several single-nucleotide polymorphisms in the NLRP3 region associated with hypoproduction of IL-1β and decreased *NLRP3* expression are associated with susceptibility to Crohn’s disease (54), which is a strong risk factor for colon cancer. In addition, individuals with polymorphisms in *NLRP3*, and *caspase 1* have a greater risk of gastric cancer when they are infected with *Helicobacter pylori*, displaying the interplay between genetic and environmental factors in tumorigenesis (55). Further evidence from cancers with virus-triggered etiology and inflammasome genetics in susceptibility to cancer development suggests that the NLRP3 inflammasome may have a protective role in virus-associated cancers (24, 56). Though, further investigations are needed to solidate this hypothesis. A dysregulated inflammasome signaling and dysbiosis both affect intestinal inflammation and cancer development, accentuating that in addition to genetic factors, environmental factors such as diet influence the ecology of the gut microbiota, inflammasome activation, and cancer (52).

Related to heart failure, a phase 1B trial with the specific NLRP3 inhibitor dapansutrile (OLT1177) has been completed in patients with stable systolic heart failure (https://clinicaltrials.gov/ct2/show/NCT03534297, accessed October 2021). The non-specific NLRP3 inhibitor, colchicine, a microtubule destabilizer traditionally used for the treatment and prevention of gouty arthritis, has been shown to exert anticancer effects *in vitro* and in animal models. In addition, colchicine decreased the risk of incident all-cause cancers in male patients with gout (57). A pilot trial of colchicine in urothelial cancer and other solid tumors is ongoing (NCT05279690). Colchicine is also first-line therapy for first and recurrent pericarditis. Its cardiobeneficial effect is and has been explored in different clinical trials ranging from acute MI (58) over stable coronary artery disease (59) to stable systolic heart failure (60). The benefit of colchicine in community-treated patients with PCR-proven COVID-19 advocates its use in those at risk of complications like myocarditis (61). Three clinically approved biologics for blocking IL-1, of which none of the 3 have an indication for CVD at the present time: canakinumab, anakinra, and the soluble chimeric Fc fusion protein of IL-1R1 and IL-1R3, rilonacept, have been and are currently under evaluation in trials over the wide range of CVD. The IL-6 (with IL-6 being downstream IL-1) antagonist, tocilizumab, which blocks soluble and membrane‐bound IL‐6R, exerts beneficial effects in a high‐risk population (rheumatoid arthritis patients), even as it increases total cholesterol and low‐density lipoprotein levels (62). Its potential has also been demonstrated for refractory severe immune checkpoint inhibitor associated myocarditis (63). Reduction in biomarkers of inflammation and thrombosis relevant to atherosclerosis has been shown in individuals with chronic kidney disease and elevated levels of C-reactive protein, following treatment with the novel IL-6 ligand inhibitor, ziltivekimab (64). The NLRP3 inflammasome activator, S100A9 has been identified as a promising biomarker and therapeutic target for different cancers (19, 65, 66) as well as for MI (67) and myocarditis (18, 68), accentuating the relevance of evaluating the potential of anti-S100A9 compounds in clinical studies of cancer (19) and heart failure (69).

### CD20 B cells and anti-CD20 therapies

The chimeric mouse/human CD20-targeting monoclonal antibody (mAb) rituximab (RTX), the first therapeutic antibody approved for oncology patients, has since its initial approval in 1997, improved the prognosis of various B cell malignancies (70). About one million patients worldwide are given anti-CD0 antibodies such as RTX for the treatment of B cell-associated diseases. In clinical practice, CD20 depleting agents are not only approved for B cell-related cancers, but also increasingly used on- and off-label for autoimmune diseases, such as rheumatoid arthritis, multiple sclerosis and systemic lupus erythematosus (71). Though, RTX in patients with hematological cancers and autoimmune disease has been associated with both atrial and ventricular arrhythmias (72) and acute myocardial ischaemia (73). Its use in CVD is untested and currently contraindicated.

The transmembrane phospholipid protein CD20, which appears on surface in the physiological maturation from pre-B to mature B lymphocytes, is also expressed on B cell-derived malignancies. Anti-CD20 mAb acts by depleting normal and malignant B cells. Anti-tumor activity of anti-CD20 has been attributed to 4 main mechanisms: antibody-dependent cellular toxicity, complement-dependent cytotoxicity, antibody-dependent phagocytosis, and FcR-dependent mechanisms. Though, despite two decades of clinical use, there is still incomplete understanding of the mechanisms behind RTX efficacy, and the biological function of CD20. Part of the complexity is the importance of the cellular microenvironment and circulatory dynamics of B cells in the efficiency of CD20 mAb-directed therapies (74). In non-B cell derived cancers, presence of CD20+ B cells in tertiary lymphoid structures around tumors is predictive of improved cancer outcome and response to checkpoint blockade (75, 76). It is suggested that the B cells might contribute to the anti-tumor response by producing antibodies against the tumors (76), or they express regulatory potency, but further studies are needed to understand the specific anti-tumor mechanism. Nevertheless, this finding addresses the dichotomous role of CD20+ B cells depending of the microenvironment and immune context, accentuating the complexity of translating CD20+ immunotherapies in cancer.

Beyond their role in cancer, there is accumulated evidence that B cells, both directly (by differentiating into plasma cells and secreting antibodies) and indirectly (by antigen presentation and cytokines/chemokines secretion), play an essential role in the progression of atherosclerosis and heart failure (77–79). Several subsets of B cells exist which differentially affect atherosclerosis (80). B1 (B1a and B1b) cells are considered atheroprotective *via* their release of primarily IgM natural antibodies against oxidation-specific epitopes that block the uptake of oxidized LDL by macrophages, preventing foam cell formation and facilitating the clearance of apoptotic cells (81, 82). In contrast, B2 cells (marginal zone and follicular B cells) are proatherogenic *via* the release of proatherogenic (auto)antibodies (79, 83). B regulatory cells have been reported to protect against atherosclerosis *via* inducing immunosuppressive T regulatory cells (84), but their importance remains controversial (85). Innate response activator B cells exert proatherogeneic effects by promoting myeloid activation (80, 86). In mice, CD20-mediated B cell depletion affects predominantly B2 cells, while B1 cells are relatively maintained (87), and is atheroprotective (79, 83). In patients, RTX-treatment has been associated with reduced endothelial dysfunction (88), decreased intima-media thickness (89) and lower arterial stiffness (90).

In the heart, anti-cardiac autoantibodies contribute directly to cardiac injury by functional or cytotoxic effects following target cell binding as well as indirectly by the formation of antigen-antibody complexes and related complement activation and inflammation [for review (77)]. Experimental evidence illustrates the involvement of mature B lymphocytes in the mobilization of inflammatory monocytes into the heart after acute MI in mice, leading to increased infarct size and deterioration of cardiac function (78). In frame with the shown protective effect of RTX in this experimental acute MI setting, an early phase experimental medicine trial, recently demonstrated safety and feasibility of a single infusion of RTX given acutely in patients with ST-elevation MI (STEMI) (91) i.e. targeting the initial inflammatory phase of damage seen in acute MI. In patients with dilated cardiomyopathy, the frequency of TNF-α-secreting B cells is increased and positively correlates with procollagen type III (92). In chronic states, RTX has been successfully applied in a case series of patients with inflammatory cardiomyopathy (93), and improved survival in cardiac allograft patients with antibody-mediated rejection (94). Though, accelerated allograft vasculopathy with RTX after cardiac transplantation has also been demonstrated (95). Safety of RTX is currently evaluated in a phase II, single-centered, single group, prospective clinical trial in stable patients with functional class III/IV according to the NYHA classification with HFrEF with an inadequate response to treatment (96).

## (Repurposed) use of advanced therapies

CAR T cell therapy has achieved durable clinical responses in patients with CD19-expressing refractory and relapsed B cell malignancies and CD269 (B-cell maturation antigen (BCMA))-expressing multiple myeloma cells and is increasingly investigated as a therapeutic option of other malignancies (97). Despite their clinical success, the use of CAR T cells can result in significant toxicities that are directly associated with the induction of powerful immune effector responses. This includes the induction of a potentially life-threatening cytokine release syndrome, which can lead to cardiovascular manifestations as tachycardia, hypotension, reduced ejection fraction and cardiogenic shock. Pretreatment with anti-inflammatory drugs, like anti-IL-6R mAb, new gene-editing technologies of *ex vivo* CAR T cell generation decreases this risk. In addition, next-generation of designed bispecific CD3-engager antibodies, targeting endogenous T cells to a defined target cell with high efficiency but limited side effects (98) opens new opportunities to use in cancer and non-cancer diseases without enhanced risk of CVD or even to treat CVD/heart failure by immune targeting (99).

Intriguingly, cardiac fibroblasts are the main source of NLRP3 inflammasome activity in the heart (100), whereas the NLRP3 inflammasome in cancer-associated fibroblasts links tissue damage with inflammation in breast cancer progression and metastasis (101). The raising relevance of fibroblasts as inflammatory supporter cells, together with their well-recognized importance as extracellular matrix-producing cells in both heart failure and cancer (102), identify fibroblasts as potential novel target cell in advanced therapies counteracting heart failure and cancer. Therefore, selective targeting “inflamed” fibroblasts would be a major breakthrough. Very recently, Rurik et al. designed a highly innovative immunotherapy strategy to generate transient CAR T cells that can recognize the fibrotic cells in the heart by *in vivo* RNA-delivery technology. Analysis of a mouse model of heart failure revealed that the approach was very successful in reducing fibrosis and restoring cardiac function (103).

## Lessons from and opportunities for repurposed use of anti-IL-1ß and anti-CD20 therapies

Lessons from NLRP3/IL-1ß and CD20 cells in cancer and CVD indicate their complexity in the pathogenesis of both separate disease entities. Their contribution to the pathogenesis is time- and context-dependent, depending on the microenvironment and immune contexture, and for NLRP3/IL-1ß may even be cell-dependent. The dichotomous response of IL-1ß and CD20 cells on cancer and CVD/heart failure addresses the difficulty of immunotherapies within each field and of translating immunotherapies from one field to the other. On the other hand, findings from the CANTOS study illustrating how IL-1ß antagonism may reduce cardiovascular events and the incidence of lung cancer underscore the possibility of killing two birds with one stone. Though, further studies are needed to clarify whether, based on the current knowledge related to the involvement of the NLRP3 inflammasome in cancer progression and cancer-therapy (doxorubicin, CAR T cells, checkpoint inhibitors,…)-related cardiac toxicity and side effects, this statement also accounts for anti-IL-1ß drugs as combination therapy with an anti-cancer treatment. The double anticancer and cardioprotective effect of anti-IL-1ß drugs let raise the hypothesis that combination of an anti-IL1ß drug with an anti-cancer therapy will allow to decrease the dose of the primary anti-cancer drug and herewith related deleterious effects. Therefore, further preclinical investigations are needed evaluating both the anti-cancer effect and the cardioprotective potential of the adjuvant anti-IL-1ß drug *via* the use of translational models, i.e. tumor-bearing mice treated with the anti-cancer drug. A large limitation of studies exploring the protective effect of anti-IL-1ß on doxorubicin-induced cardiotoxicity so far was that mainly non-tumor bearing mice were used, lacking the contribution of the tumor-associated inflammation on cardiac dysfunction. Furthermore, many studies were/are often directed at exploring only one of both aspects, be it the oncologist investigating the anti-cancer aspect and the cardiologist evaluating the cardioprotective effect of the drug, underscoring the necessity of interdisciplinary investigations.

## Biomarker-driven stratification

Learning from precision medicine in oncology and the disappointing results from past clinical trials with anti-inflammatory therapies in CVD and heart failure reflecting the diversity of inflammation in those patients (69), has led to the recognition of the need for patient stratification to better guide anti-inflammatory/immunomodulatory therapeutic interventions in the cardiology field (104). In fact, the outcome of the CANTOS trial (14), is partly built on the specific inclusion of post-MI patients with a residual inflammatory risk mirrored by high sensitive C-reactive protein levels, reflecting high IL-1ß levels (105), and their hereto treatment with the IL-1ß antibody canakinumab. Another example illustrating the power of biomarker-driven therapies, here the treatment of patients with a specific immune profile with a hereto-connected immune cell-targeted strategy, follows from virus-negative inflammatory cardiomyopathy patients with endomyocardial biopsies positive for CD20-positive B cells. Evidence from a case series illustrated that the clinical course of those patients, refractory to a classical immunosuppressive therapy with prednisolone and azathioprine, improved following repurposed treatment with the anti-cancer drug RTX (93).

Despite the ability to identify and quantify specific immune cell subsets in endomyocardial biopsies of patients with suspected myocarditis/inflammatory cardiomyopathy, aetiology-specific therapies for myocarditis/inflammatory cardiomyopathy are still in their infancy (106, 107). This might be partly explained by the tools used to diagnose myocarditis/inflammatory cardiomyopathy, which is – following the ESC guidelines – based on the quantification of CD3 and CD68/Mac-1 infiltrating cells in endomyocardial biopsies *via* immunohistochemistry (≥ 14 leukocytes/mm^2^ including up to 4 monocytes/mm^2^ with the presence of CD3 positive T-lymphocytes ≥ 7 cells/mm^2^) (108). This classical diagnostic work-up does not differentiate between T cell subsets (e.g. Treg/Teff), nor between pro- and inflammatory monocytes. This calls for a further defined and standardized evaluation of immune cells subtypes, gene expression profiles, or imaging to better mirror the cardiac immune homeostasis in those patients, allowing better patient stratification and differentiation of the stage in the pathogenesis. In this regard it is important to address that immune signatures (109) or ratios (Treg/Teff; pro-/anti-inflammatory monocytes) better reflect the immune status, not being restricted to only one specific marker or target, which due to redundancy of inflammation may be compensated *via* other inflammatory signaling pathways. Novel technologies initially used in the (immuno)-oncology field, including single cell (nucleus) sequencing, multiplex immunofluorescence, and mass cytometry may here be of value to close the current gaps related to the diagnosis of myocarditis based on immunohistochemistry. This does not imply their integration *per se* in daily diagnostic procedures. Though, screening of clinical samples *via* those state-of-the art techniques may identify novel diagnostic targets, which may then be integrated in routine diagnostic procedures.

## Conclusion

**Figure 1:**
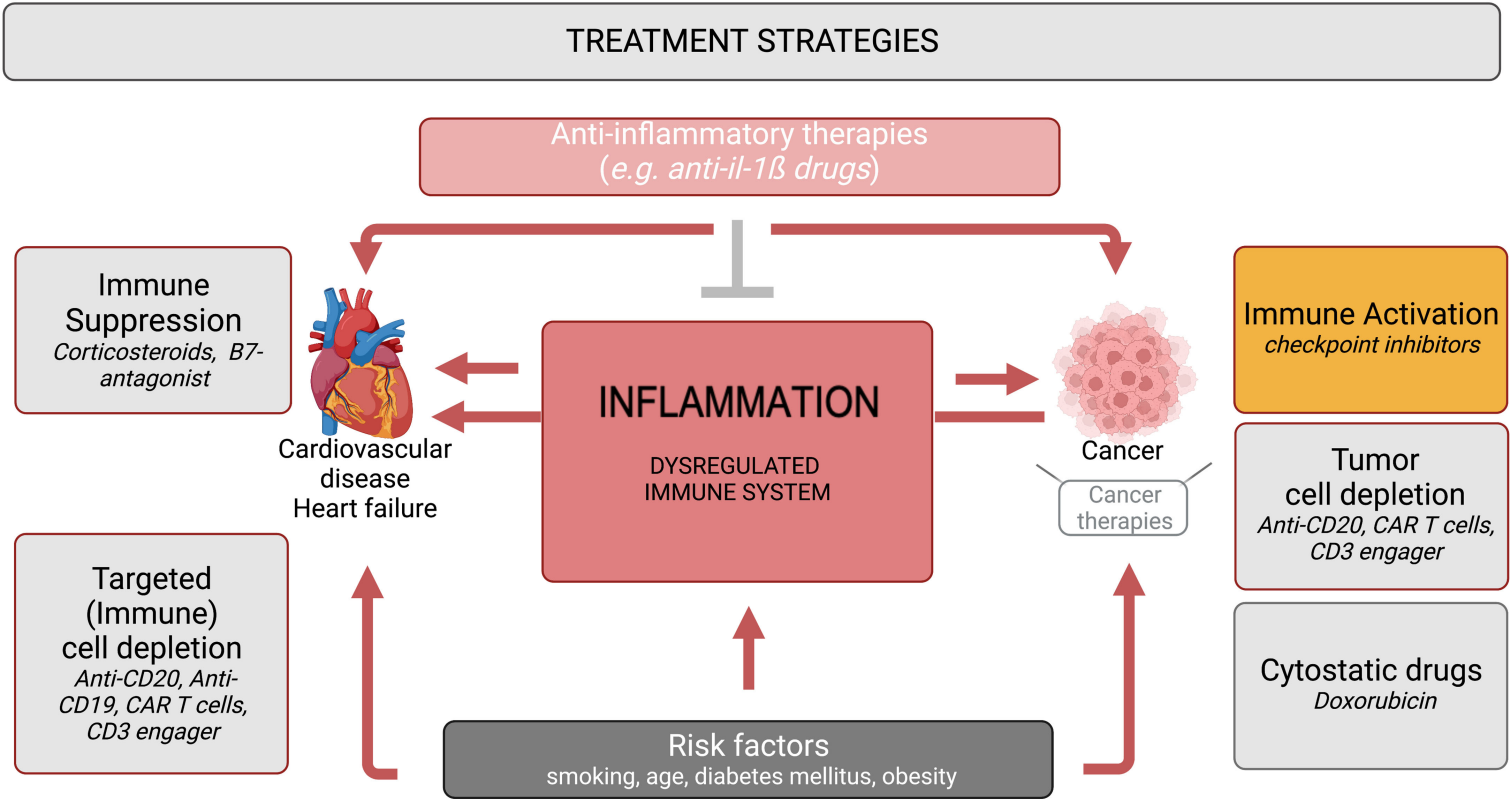
Immuno-cardio-oncology – anti-inflammatory and immunomodulatory strategies in CVD/heart failure and cancer. Inflammation and a dysbalanced immune system, provoked by risk factors such as smoking, age, diabetes mellitus and obesity, are common triggers in the pathogenesis of cancer and CVD/heart failure. Anti-cancer therapies comprise strategies directed to activate the immune response e.g. checkpoint inhibitors, to deplete tumor cells (anti-CD20, CAR T cells, CD3 engager), and cytostatic drugs e.g. doxorubicin, of which checkpoint inhibitors, CAR T cells and doxorubicin provoke cardiac inflammation. In contrast, immunosuppressive therapies like corticosteroids and B7-antagonists are used for the treatment of CVD and heart failure. Repurposed (immune) cell depletion strategies (anti-CD20, CD19 antibodies, CAR T cells, CD3 engager) have entered the cardiology field. Anti-inflammatory therapies (e.g. anti-IL-1ß drugs) are under investigation for the treatment of cancer, cancer therapy-related inflammation and CVD/heart failure.

Deeper understanding of the interaction between inflammation, cancer, CVD/heart failure – addressed in immuno-cardio-oncology – opens new options for preventing negative effects of cancer and cancer therapies on the heart on the one hand and for repurposing novel targeted therapeutic options and concepts from the cancer field for treating CVD (or *vice versa*) on the other hand (**Figure 1**). Hereto, further investigations are needed to disentangle context- and tissue-specific inflammation among the diversity of cancer types and CVD/heart failure.

## Author contributions

SVL and H-DV wrote the manuscript. SVL concepted the manuscript. All authors revised the manuscript for intellectual content and gave their final approval for publication.

## Conflict of interest

The authors declare that the research was conducted in the absence of any commercial or financial relationships that could be construed as a potential conflict of interest.

The handling editor SS declared a shared parent affiliation with the authors at the time of review.

## Publisher’s note

All claims expressed in this article are solely those of the authors and do not necessarily represent those of their affiliated organizations, or those of the publisher, the editors and the reviewers. Any product that may be evaluated in this article, or claim that may be made by its manufacturer, is not guaranteed or endorsed by the publisher.
